# A multidisciplinary approach to the management of disorders of gut-brain interaction: psychopharmacology, psychotherapy, and diet

**DOI:** 10.3389/fgstr.2025.1637172

**Published:** 2025-10-07

**Authors:** Francisco J. Barrera Flores, José Adrián Guerrero Tamez, Tatiana Winkelman, Regina Barrera Flores, Elizabeth N. Madva

**Affiliations:** ^1^ Department of Psychiatry, Massachusetts General Hospital, Harvard Medical School, Boston, MA, United States; ^2^ Department of Psychiatry, Hospital Universitario “Dr. José Eleuterio González”, Universidad Autónoma de Nuevo León, Monterrey, Mexico; ^3^ Department of Nutrition, Brigham and Women’s Hospital, Harvard Medical School, Boston, MA, United States

**Keywords:** DGBI, psychotherapy, psychopharmacology, IBS, neuromodulators

## Abstract

**Introduction:**

Disorders of gut-brain interaction (DGBI), including irritable bowel syndrome and functional dyspepsia, are chronic gastrointestinal syndromes characterized by visceral hypersensitivity and altered brain-gut signaling in the absence of known structural pathology. A significant proportion of individuals with DGBI have comorbid psychiatric conditions, especially anxiety and depression, highlighting the biopsychosocial underpinnings of these disorders.

**Methods:**

This narrative review synthesizes the neurophysiological, psychological, pharmacological, and psychotherapeutic literature related to DGBI. We examined the role of gut-brain axis dysregulation, the prevalence and impact of psychiatric comorbidity, and evaluated current treatment modalities, including neuromodulators, brain-gut behavior therapies (BGBTs), and dietary interventions.

**Results:**

Neuroimaging and genetic studies support the role of emotional and cognitive circuits in modulating gut sensitivity and symptom perception. Psychiatric comorbidity, particularly anxiety, is bidirectionally linked to DGBI and influences treatment response. Neuromodulators such as tricyclic antidepressants demonstrate modest efficacy. BGBTs—including cognitive behavioral therapy and gut-directed hypnotherapy—exhibit comparable efficacy to pharmacologic treatments, with sustained symptom relief and additional benefit on mood and illness-related beliefs.

**Discussion:**

DGBI represent complex, stress-sensitive conditions best managed through multidisciplinary care. Integration of pharmacologic neuromodulation, psychotherapeutic interventions, and dietary strategies targeting the brain-gut axis offers the most comprehensive approach. Future research should refine treatment matching based on symptom phenotype, psychological profile, and gut-brain biomarkers to improve long-term outcomes.

## Introduction

Dysregulation of the brain-gut axis, or the bidirectional communication system between the central and enteric nervous systems, is the core feature of the disorders of gut-brain interaction (DGBI), a subset of gastrointestinal conditions. The DGBI are syndromes defined by a set of clinical features and symptoms, without demonstrable pathology on testing or biomarkers that enable diagnosis. Conceptualized as stress-sensitive, biopsychosocial disorders, it is estimated that approximately half of all patients with DGBI have a comorbid psychiatric condition, with anxiety disorders being the most common. In this review, we describe the pathophysiology of these conditions, as well as the psychopathological profile often associated with their presentations, and discuss the evidence supporting the benefit of pharmacologic, psychotherapeutic, and dietary interventions for these patients.

## Methods

To inform our narrative review, we developed five structured PubMed search strategies targeting key thematic areas related to disorders of gut-brain interaction (DGBIs): neurophysiological mechanisms, psychological comorbidity, pharmacologic treatment, psychotherapy, and dietary interventions (see **Appendix 1**). Each search string included both umbrella terms (e.g., “functional gastrointestinal disorders,” “IBS,” “functional dyspepsia”) and topic-specific keywords (e.g., “brain-gut axis,” “anxiety,” “SSRIs,” “CBT,” “FODMAP”) as well as filters for publication date (1995–present), English language, human adults, and title/abstract field limits to improve specificity. These searches were designed to identify the most representative, conceptually illustrative, clinically meaningful, and high-yield literature relevant to each domain. Final article selection from each search was guided by the authors’ clinical experience and research expertise.

## Neurophysiological basis of gastrointestinal disorders (brain-gut interaction)

The “brain-gut axis” is a bidirectional communication system between the central nervous system (CNS) and the enteric nervous system (ENS). This system signals homeostatic information to the brain through neural (spinal and vagal) and humoral pathways, influenced by the gut microbiome and relying on immune, endocrine, neural, and metabolic pathways (1, 2). Dysregulation of this axis is the core feature of disorders of the DGBI (3).

Visceral pain arises from the conscious perception of gut-brain signals induced by noxious stimuli. These signals are processed in a homeostatic-afferent network (brainstem sensory nuclei, thalamus, posterior insula) and modulated by emotional (amygdala, anterior cingulate cortex) and cognitive circuits (prefrontal cortex, anterior insula). Descending projections modulate pain at the spinal level. Dysfunction in these systems is believed to contribute to visceral hypersensitivity, a hallmark of the DGBI (4). Attempting to elucidate specific areas in the brain associated with this visceral hypersensitivity, structural MRI studies in individuals with IBS have identified some distinctions. These include increased gray matter volume in the primary and secondary somatosensory cortices and subcortical areas, as well as reductions in the posterior insula and superior frontal gyrus (5). Greater cortical thickness in the primary somatosensory cortex has been associated with higher pain intensity ratings during rectal distension, while larger nucleus accumbens volume correlates with lower rectal pain thresholds, suggesting that repeated visceral pain may drive neuroplastic changes in somatosensory and reward-related regions (5). Additional structural abnormalities have been reported in the insular cortex, where reduced cortical thickness and gray matter volume are linked to longer symptom duration and heightened visceral sensitivity (6). Functional neuroimaging further supports the insula’s role as an integrative hub, showing increased activation during rest and in response to visceral stimuli, such as colorectal distension, in both irritable bowel syndrome (IBS) and animal models (7).

Compared to healthy populations, patients with IBS have increased brain activity in homeostatic-afferent regions (8), and individuals with functional dyspepsia (FD) fail to deactivate the amygdala during anticipated gastric distension, which is correlated with higher levels of anxiety (9). Anxiety-related impairment of the descending modulatory system may contribute to why physiological gastric distension is perceived as painful (4).

Animal studies have further clarified the insula’s role in modulating pain: reducing the excitability of pyramidal neurons within this region attenuated both visceral hypersensitivity and anxiety-like behavior associated with pain, highlighting the insular cortex as a potential therapeutic target for DGBIs (10). The neural pathway connecting the insular cortex and the nucleus tractus solitarius (NTS) represents a core component of brain-gut communication. Afferent signals from the gastrointestinal tract travel via the NTS to the insula, influencing central sensitization by encoding visceral discomfort. Descending projections from pyramidal neurons in the insula can influence the medullary vagal complex, thereby modulating gastrointestinal motility and contributing to peripheral pain sensitization (11). This bidirectional circuit may underlie the interplay between emotional regulation and somatic symptom perception in functional gastrointestinal disorders.

There is evidence that diet also plays a key role in DGBI symptomatology; food form and nutrient composition may trigger GI symptoms through various mechanisms, including bacterial fermentation altering gut microbiota, osmotic effects in the intestines, gas production, and immune responses (12). Several microbial- neural mechanisms have been implicated in visceral pain modulation. For instance, both Lactobacillus reuteri supplementation and pharmacologic blockade of IK(Ca) channels have produced similar changes in colonic motility and excitability of myenteric neurons in rodent models, suggesting a shared pathway influencing pain perception (13). In addition, TRPA1 receptors, abundantly expressed in the nodose ganglia (the inferior sensory gangial of the two vagus nerves), act as detectors of chemical irritants and contribute to the neurobiology of inflammatory pain. There is emerging speculation that damage to the intestinal epithelium may allow bacteria such as *Edwardsiella tarda* to directly activate TRPA1 in the nodose ganglia, potentially initiating neuroplastic changes in the central nervous system and contributing to disease pathogenesis (13–15).

Finally, a recent review of the literature identified three principal routes through which gut microbiota imbalances may influence brain function. First, the vagus nerve serves as a key conduit for signaling between the gut and central nervous system. Second, increased intestinal permeability may allow microbial products to cross into systemic circulation. Finally, certain bacterial metabolites, such as lipopolysaccharides, can trigger neuroinflammatory responses and alter neurotransmitter systems, potentially contributing to cognitive and affective symptoms seen in DGBIs (16).

## Psychological comorbidity in gastrointestinal diseases

Psychological comorbidity in gastrointestinal disorders is often misconstrued as being confined to DGBI or as merely a consequence of physical illness (17). Psychological symptoms, however, are not only prevalent in DGBI but are often integral to their development and maintenance (4). A bidirectional two-sample Mendelian randomization study demonstrated that both genetically predicted depression and genetically predicted anxiety were significantly associated with an increased risk of several DGBI. In this context, these psychiatric traits were estimated based on genetic variants identified through large-scale genome-wide association studies (GWAS), which served as instrumental variables to infer potential causal effects while minimizing confounding factors. Specifically, genetically predicted depression was linked to a higher risk of functional dyspepsia (FD), IBS, and functional constipation (FC), while genetically predicted anxiety was associated with an increased risk of IBS (18).

A growing body of evidence supports a high psychological burden among the DGBI. Much of the evidence comes from studies examining the DGBI that are relatively more prevalent (e.g., IBS, FD), as there has been greater research on these. In IBS, for example, a multivariate analysis of 769 patients demonstrated that 44.9% reported significant anxiety, while 25.7% met criteria for depression (19). Furthermore, a large-scale study involving 158,565 cases and 300,995 controls in the United Kingdom identified that stress, anxiety, and depression are potential underlying etiological factors for IBS; individuals with these factors have higher odds of having IBS (OR: 1.06 (95% [C.I]: 1.03-1.08) (20).

Panic disorder is also prevalent in IBS, with studies indicating that close to 55% of IBS patients meet diagnostic criteria for this condition (21).Beyond mood and anxiety disorders, psychological concerns in IBS often include maladaptive cognitive patterns, such as catastrophizing, illness anxiety, and hypervigilance (22). Gastrointestinal-specific anxiety has been identified as a significant mediator between general anxiety symptoms and IBS symptom severity (22–24). Additionally, somatization has been associated with IBS, particularly the mixed subtype (IBS-M), with one study reporting that 32% of these patients exhibited this form of psychological distress (25).

FD is believed to share similar mechanisms with IBS, particularly visceral hypersensitivity, manifested as discomfort with distention of the gastric fundus during meals and even in resting states (26). Numerous studies have shown higher rates of anxiety and depression in individuals with FD, however, comorbidity estimates vary significantly (27). Of the psychiatric disorder, anxiety may have a unique mechanistic role in the development of FD. For instance, a 10-year follow-up study of 887 participants in Sweden found that anxiety at baseline was associated with new-onset FD, but no such association was observed for depression (28). Additionally, a phenotypic and genetic cross-disease analysis involving 10,078 cases identified anxiety disorders as one of the diagnoses most strongly associated with FD (29).

Functional constipation (FC), another DGBI, differs from IBS in that abdominal pain is not a requirement for diagnosis. Similar to IBS, anxiety and depression are more prevalent in patients with FC compared to the general population (30). A recent study involving elderly men and women found that 30% of patients with FC also had depression, while 21% also had anxiety (31). Preclinical research has also implicated serotonin production in the pathogenesis of both constipation and depression, highlighting potential shared pathophysiology and targets for treatment (32) (23, 33–40). In sum, systematic assessment of psychological comorbidities among individuals with DGBI is an important step in the formulation of an effective and comprehensive treatment plan.

## Pharmacology

Pharmacologic treatment approaches, including the use of “neuromodulators” or medications targeting the gut-brain axis, to treat GI symptoms and gut motility are increasingly being incorporated into clinical practice. The term neuromodulator refers to medications including antidepressants, antipsychotics, and antiepileptics that exert their effects on the gut-brain axis through serotoninergic, noradrenergic, and dopaminergic pathways (41, 42). Neuromodulators may be particularly useful or relevant for patients with DGBI with comorbid anxiety or depression, though not exclusively. It has been noted that patients with comorbid anxiety are more likely to be treated with a neuromodulator by their gastroenterologist, but paradoxically, may also be less likely to demonstrate the desired treatment response to either neuromodulators or any medication prescribed for their gastrointestinal symptoms. This finding highlights the complex relationship between anxiety and gastrointestinal function, which currently available medications may not adequately or sufficiently target (42). This finding may also reflect what is often observed in clinical practice, that patients with anxiety tend to demonstrate greater sensitivity to their physical sensations, and thus may have more difficulty identifying medications that they can reliably tolerate.

Neuromodulators are increasingly being prescribed for patients with DGBI. A U.S study found that over half (55%) of all gastroenterologists agreed that neuromodulators were key to the management of IBS, for example, with the majority favoring tricyclic antidepressants, though the side effect burden was noted as a common concern (43). Evidence suggests, however, that neuromodulators are largely well tolerated, as, in at least one study, only ~10% of those receiving neuromodulators developed side effects that led to discontinuation (44). Interestingly, there is evidence suggesting that a strong nocebo effect in this patient population could predispose them to early discontinuation (44). Additionally, other studies have demonstrated that certain symptoms attributed by patients to neuromodulators existed prior to treatment and do not correspond to actual drug-related effects (45).

Further research is needed to evaluate the efficacy of neuromodulators to treat GI symptoms specifically (46, 47), though a growing body of research has examined TCAs and SSRIs. A recently published meta analysis evaluated the efficacy of antidepressants in the treatment of IBS demonstrated that those individuals taking antidepressants had three times the odds of experiencing global symptom improvement. Subgroup analyses indicated that both SSRIs and TCAs were associated with roughly two and three times the odds of global symptom improvement, respectively. Notably, this therapeutic effect was also evident in individuals who had not responded to standard initial treatments (48). These findings suggest a robust effect size, and build on earlier estimates from Ford et al. (47), in which the pooled NNT for antidepressants (including both SSRIs and TCAs) was 4.5, highlighting possible shifts in effect size estimates with the inclusion of more recent trials (46, 47). When comparing TCAs to placebo, a recent meta-analysis demonstrated that TCAs carry a relative risk of 0.66 (0.53-0.83) in failing improvement in global IBS symptoms at 4–12 weeks of treatment (49). However, these were also more likely than placebo to lead to side effects with a relative risk of 1.59 (1.26-2.06). In another study, TCAs were associated with a relative risk of 0.70 (0.62-0.80) of failing to improve global IBS symptoms and 0.69 (0.54-0.87) of failing to improve abdominal pain. SSRIs carried a relative risk of 0.74 (0.56-0.99) (50).

Relatively fewer clinical studies have examined serotonin-norepinephrine reuptake inhibitors (SNRIs) such as venlafaxine and duloxetine, in DGBI. The SNRIs, however, have empirical value based on their mechanisms of action (41). Evidence shows that venlafaxine increases colonic compliance, decreases tone, reduces postprandial colonic contractions, and reduces pain intensity ratings during graded distensions (51). SNRIs also carry a relative risk of 0.22 (0.08-0.59) of failing to improve abdominal pain (50). A recent small, retrospective study also demonstrated a likely benefit of SNRIs in targeting symptoms of bloating, though further research is needed (52).

Early research also indicates a likely benefit to other neuromodulators, such as buspirone and gabapentin, in the management of DGBI. In a recent meta-analysis, for example, buspirone improved bloating severity more than placebo but did not improve postprandial fullness or nausea severity (53). Low doses of gabapentin, on the other hand, can improve symptoms of functional dyspepsia, particularly postprandial fullness, upper abdominal pain, and nausea and vomiting (54). Selected studies outlining the evidence for neuromodulators in the management of DGBIs are outlined in **Table 1**.

**Table 1:** Select studies of psychopharmacological interventions in DGBIs.

Study	Study design	Sample size	Active arm	Comparator	Primary endpoint	Main findings
Ford, 2023 (96)	Placebo controlled RCT	463 adults with IBS	Amitriptyline, 10 to 30mg daily	Placebo	IBS-SSS score at 6 months	Amitriptyline group with significantly decreased IBS-SSS score as compared to placebo (-27.0 [-46.9 to -7.1]; p=0.0079)
Agger, 2017 (97)	Placebo controlled, double blind RCT	120 adults with multiorgan bodily distress syndrome	Imipramine, 25 to 75mg daily	Placebo	Self-reported overall health improvement on 5 point CGI scale	OR 3.3 (1.6-6.8) favoring treatment with imipramine
Ladabaum, 2010 (98)	Placebo controlled, double blind RCT	54 adults with IBS	Citalopram 20mg daily	Placebo	Overall IBS symptom score	No difference between citalopram and placebo (d=0.398)
Houghton, 2025 (99)	Placebo controlled, double blind RCT	308 adults with IBS	Novel alpha-2-delta ligand	Placebo	adequate relief of abdominal pain/discomfort for ≥ 50% of the active treatment period	Novel medication no more effective than placebo (OR 0.96 [0.65, 1.41])
Cheong, 2018 (100)	Placebo controlled, double blind RCT	107 adults with functional dyspepsia	Imipramine 50mg daily	Placebo	Self-reported satisfactory relief of global dyspepsia symptoms at 12 weeks	Imipramine more effective than placebo at providing relief of global dyspepsia symptoms, with NNT = 4
Talley, 2015 (101)	Placebo controlled, double blind RCT	292 adults with functional dyspepsia	Amitriptyline 50mg daily and escitalopram 10mg daily	Placebo	Self-reported adequate relief of symptoms at least 50% of the 10 treatment weeks	Amitriptyline had greater rate of relief of symptoms as compared to placebo (OR 2.1[1.04, 4.36]). No difference for those taking escitalopram as compared to placebo
Tack, 2012 (102)	Placebo controlled, double blind RCT crossover	17 adults with functional dyspepsia	Buspirone 10mg TID before meals	Placebo TID before meals	Self-reported dyspepsia symptom severity (DSS) score	Significantly lower DSS following buspirone, but not placebo, treatment
Sharbafchi, 2023 (103)	Placebo controlled, double blind RCT	37 adults with IBS	Duloxetine 60mg daily	Placebo	Mean IBS-SSS score at 10 weeks of treatment	Duloxetine group with lower IBS-SSS scores at week 10 as compared to placebo
Staller, 2019 (54)	Retrospective, open-label, observational study	62 adults with functional dyspepsia	Gabapentin, dose range not specified however median ending daily dose 954mg ± 1184 mg	N/A	Change in PAGI-SYM score (with -0.3-point decrease being significant)	Mean decrease in PAGI-SYM score -0.44 points

Patient Assessment of Gastrointestinal Disorders-Symptom Severity Index (PAGI-SYM).

Irritable Bowel Syndrome Symptom Severity Scoring System (IBS-SSS).

Dyspepsia symptom severity (DSS).

Clinical Global Improvement (CGI).

### Side effects of pharmacotherapy

Each neuromodulator group is associated with common side effects that clinicians should monitor regularly. TCAs frequently cause sedation, dry mouth, constipation, weight gain, and orthostatic hypotension, particularly at higher doses (55). SSRIs and SNRIs often lead to nausea, insomnia or somnolence, sexual dysfunction, and gastrointestinal disturbances (56). Gabapentinoids, are commonly associated with drowsiness, dizziness, ataxia and rare cases self-harm behaviors (57, 58).

While these side effects are generally manageable, clinicians should also be aware of serious adverse events, especially in vulnerable populations. TCAs have been associated with cardiotoxicity, including QRS/QT prolongation and ventricular arrhythmias due to sodium-channel blockade, even at therapeutic doses (59). A systematic review found that amitriptyline, nortriptyline, and clomipramine carry measurable cardiac risks even with routine use (60).

SSRIs and SNRIs increase the odds of hyponatremia (OR = 3.16; 95% CI 1.91–5.23), with event rates of 7.4% for SNRIs, 5.6% for SSRIs, and 2.7% for TCAs. Evidence suggests this risk is more pronounced during the first two weeks of treatment, particularly among elderly patients or those on diuretics (61).

Gabapentin and pregabalin carry a recognized risk of misuse. They are frequently used to enhance opioid effects or self-manage anxiety, insomnia, or withdrawal, often leading to dose escalation, loss of control, and psychiatric withdrawal symptoms (62). Misuse rates range from 1.6% in the general population to over 60% among individuals with opioid use, with motivations including euphoria and deliberate intoxication (63, 64).

## Psychotherapy

Many psychotherapeutic approaches, or brain-gut behavior therapies (BGBTs), have also demonstrated efficacy in reducing the symptom burden of patients with DGBIs. BGBTs are short-term, non-pharmacologic interventions that have been adapted from traditional psychotherapies to specifically target gastrointestinal symptoms, though they may also benefit psychological comorbidity (65). Of the BGBTs currently available, cognitive-behavioral therapy (CBT) and gut-directed hypnotherapy have been the most studied and thus have the most evidence (47).

Most studies examining the efficacy of BGBTs have been completed in individuals with either IBS or FD, given their higher prevalence, though expert consensus is that future research will likely demonstrate efficacy of the BGBTs for other DGBI as well (65). A recent meta-analysis demonstrated that the number needed to treat (NNT) for CBT for IBS is approximately 4, and the NNT for gut-directed hypnotherapy for IBS is approximately 5 (47). Notably, this is comparable to the NNT of 4.5 for antidepressants in patients with IBS.

Compared to CBT and gut-directed hypnotherapy, relatively fewer studies have examined other BGBTs such as exposure-based therapy, mindfulness, disease self-management programs, or psychodynamic psychotherapy, among others. Studies evaluating both CBT and hypnotherapy have yielded promising results, and these will be summarized in subsequent sections. Though fewer studies have examined these other types of BGBTs, a growing body of evidence indicates a likely benefit to their use. For instance, a systematic review and meta-analysis of randomized controlled trials (RCTs) of individuals with IBS suggested that mindfulness, compared to control, was associated with improvement in symptom severity, pain, and quality of life (66). An RCT comparing acceptance and commitment therapy (ACT) to dialectical behavioral therapy (DBT) and mindfulness based stress reduction (MBSR) in individuals diagnosed with IBS demonstrated that the ACT intervention was associated with lower levels of IBS symptoms, anxiety, and depression, as well as higher quality of life when compared to the other groups (67). Finally, based on two RCTs, psychodynamic psychotherapy appears to lead to improvement in IBS symptoms with a NNT of 4 (47).

Further research is needed to elucidate the mechanisms of BGBTs. Early mechanistic work has explored the role of “psychological” factors, such as self-efficacy, a positive treatment expectancy for symptom improvement, or patient-therapist bonding, as potential mediators of treatment (68), as well as a variety of “biological” factors such as gut microbiome composition (69). In the following sections, we summarize the current evidence for gut-directed hypnotherapy and CBT for DGBIs, and selected papers are outlined in **Table 2**.

**Table 2:** Select studies of psychological interventions in DGBIs.

Study	Study design	Sample size	Active arm	Comparator	Primary endpoint	Main findings
Lindfors, 2012a (71)	RCT	90 adults with IBS	Gut directed hypnotherapy	Supportive therapy	GI symptom questionnaire at 3 months	Greater improvement in hypnotherapy group as compared to supportive therapy 3.7 (0.3–7.2); *p* = 0.03
Lindfors, 2012b (71)	RCT	48 adults with IBS	Gut directed hypnotherapy	Waitlist	GSRS-IBS at 3 months	Significant reduction in symptoms in active treatment, but no significant difference between treatment and control groups (0.33 [−0.22, –0.91], p=0.22)
Lackner, 2018 (76)	RCT	436 adults with IBS	Standard CBT and minimal contact CBT (MC-CBT)	IBS education	% participants “substantially improved” or “moderately improved” on CGI immediately after treatment	Significantly larger percentage of participants substantially or moderately improved in MC-CBT as compared to IBS education
Thakur, 2017 (104)	RCT	106 adults with IBS	Emotional awareness and expression training (EAET); relaxation training	Waitlist	Change in IBS-SSS at 10 weeks post-treatment	EAET but not relaxation training reduced IBS symptoms severity as compared to waitlist (*F* (4, 206)=2.43, *p* = .026)
Everitt, 2019 (105)	RCT	558 adults with IBS	Telephone delivered CBT and Web (TCBT) delivered CBT (WCBT)	Treatment as usual (TAU; no additional psychological therapy)	IBS-SSS at 12 months after initiation	Significantly lower IBS-SSS at 12 months in TCBT (p<0.001) and WCBT (p=0.002) as compared to TAU
Hoekman, 2021 (70)	Open-label RCT	63 adults with IBD in remission and IBS	Gut directed hypnotherapy	Standard medical treatment of various modalities	% participants with reduction of ≥50% on visual analog scale for pain at week 40	No difference between groups in proportion of participants meeting primary outcome [difference=3% [−19 to 24%], *p* = 0.81

Gastrointestinal Symptom Rating Scale IBS version (GSRS-IBS).

Irritable Bowel Syndrome Symptom Severity Scoring System (IBS-SSS).

### Hypnotherapy

We estimate that there are at least 15 RCTs comparing the effectiveness of hypnotherapy to several interventions including standard care, other forms of therapy, diet, exercise, or education. Collectively, these RCTs demonstrate favorable results for hypnotherapy for several different DGBIs and DGBI-related symptoms.

Hoekman et al., randomized 80 adult participants in an open-label study evaluating the effect of hypnosis versus standard medical treatment (following the Dutch multidisciplinary guideline for diagnosis and management of IBS) on clinical remission and biochemical remission (using fecal calprotectin) in patients with quiescent IBD plus IBS-type symptoms. The primary outcome (≥50% reduction in symptom severity measured with the IBS symptom severity scale (IBS-SSS) at week 40) was met in 30% of the patients randomized to hypnotherapy versus 27% of the ones in standard treatment, without statistical significance. Notably, adequate relief was reported in 60% *vs* 40% in hypnotherapy and standard treatment, respectively (70).

Lindfors et al., conducted a series of 2 RCTs evaluating the effectiveness of hypnotherapy on IBS. In the first study, participants were randomized to receive the hypnotherapy intervention or supportive therapy in a psychology private practice. In the second study, participants were randomized to receive the intervention or be placed on a waitlist. In both studies, IBS symptoms improved at 3 months in the hypnotherapy group but not in the control group, with effects sustained at 1-year follow-up (71).

A 2007 systematic review including 4 studies comparing hypnotherapy to psychotherapy, placebo, waitlist, or standard medical management, argued that hypnotherapy was superior to waitlist control or standard medical treatment in reducing the severity of IBS symptoms. However, they were unable to pool the data in a meta-analysis given differences in outcome measures and study design, which speaks to the methodological heterogeneity of the included studies (72).

### Cognitive behavioral therapy

At least 30 RCTs have examined CBT (most often in patients with IBS) and demonstrated its efficacy in reducing symptoms in a variety of different formats and forms of delivery. It has been also demonstrated that the benefit provided by CBT is often maintained long-term (73). For example, one study randomized patients with IBS to either weekly hour CBT sessions over 10 weeks, 4-hour sessions over 10 weeks, or wait list, and demonstrated that 30% of people receiving CBT were rapid responders and the majority of these maintained the benefit at a 3-month follow-up examination (74). Interestingly, rapid responders were more likely to have higher symptom severity (74). In another study, it was demonstrated that the benefits of using a self-administered and a therapist-administered CBT intervention were comparable (75). A large, 3-arm randomized trial allocating over 400 participants with IBS to either weekly hour-long CBT sessions, 4 home-based CBT sessions with minimal therapist contact, or 4 sessions of IBS education, demonstrated that home CBT is as effective as standard CBT and both of these interventions were slightly superior to education alone at 6 months follow up (76, 77). Furthermore, in a 12-month follow-up analysis from this same study, they concluded that over 30% of the participants receiving either version of CBT sustained the same benefits, compared to only 20% of those receiving IBS education (73). Notably, a multicenter randomized control trial found that the efficacy of CBT is similar when delivered in person or via app, lowering the barrier of delivery of these interventions (78). And a meta-analysis of app-delivered CBTs in IBS found medium to large effect sizes on IBS (79).Another study suggested that the benefits of CBT on these symptoms might be mediated by a positive treatment expectancy for symptom improvement and a good patient-therapist relationship, both early and in the long-term (68).

While BGBTs such as cognitive behavioral therapy and gut-directed hypnotherapy demonstrate efficacy comparable to pharmacologic approaches, their real-world implementation can be limited by accessibility, acceptability, cost, and availability of trained providers. In many settings, neuromodulators may be more readily accessible and affordable, which can influence both clinician recommendations and patient preferences. Acknowledging these practical barriers is essential when translating evidence into individualized treatment planning.

## Dietary interventions

Dietary interventions may also play an important role in treatment for some patients with DGBIs, particularly when implemented under the supervision of a dietician. The low fermentable oligo-, di-, monosaccharides, and polyols (FODMAP) diet is perhaps the most well-known and established dietary therapy that has been shown to reduce symptoms of IBS and other DGBIs (80). A recent meta-analysis, for example, found a moderate reduction in symptom severity (standardized mean difference -0.53; 95% CI, -0.68 to -0.38) from the FODMAP diet compared to control diets among patients with IBS (81). Interestingly, a randomized clinical trial compared hypnotherapy, the FODMAP diet, or a combination of both and assessed IBS symptom severity (82). At week 6, all three groups demonstrated similar effectiveness in reduction of symptoms with no notable differences in the effect between groups; ≥20% improvement was achieved in 72%, 71%, and 72% across groups at 6 weeks and 74%, 82%, and 54% at 6 months, respectively. Compared to the two other groups, however, hypnotherapy was superior when assessing anxiety and depression at 6 months (82).

Other dietary interventions have also demonstrated benefit for patients with DGBIs. Certain fibers, for example, can help regulate bowel movements, reduce colonic fermentation to minimize bloating, and benefit gut microbiota. When combined with the low FODMAP diet, psyllium and inulin reduce colonic gas production, and sugarcane bagasse with resistant starch shifts fermentation to the distal colon – both potentially easing GI symptoms (83). Peppermint oil capsules have also been studied and a meta-analysis demonstrated their association with a relative risk of 0.63 (0.48-0.83) for failing to achieve improvement in global IBS symptoms at 4–12 weeks of follow up. And interestingly, there were no significant differences when comparing the effectiveness of peppermint oil capsules to that of TCAs in this meta-analysis (49).

Emerging research highlights how strengthening the gut microbiota and enteric nervous system with prebiotics, probiotics and their metabolites (like tryptophan and short-chain fatty acids [SCFAs]) may improve gut-brain function. Synbiotics –combinations of probiotics (e.g., *Lactobacillus, Enterococcus*, and *Bifidobacterium*) and prebiotics (like inulin and resistant starch)– have been shown to boost neurotransmitters and neuropeptides (e.g., gamma-aminobutyric and brain-derived neurotrophic factor), improving CNS activity and psychiatric disease-related functions, such as anxiety, depression, stress, and memory (84). Furthermore, tryptophan, an essential amino acid, play a role in gut-brain signaling by influencing metabolic pathways linked to CNS inflammation. Emerging evidence suggests that microbial metabolites of tryptophan may help reduce neuroinflammation. Disruptions in tryptophan metabolism have been observed in individuals with DGBI (e.g., IBS) and neuropsychiatric conditions (e.g., depression, autism). While human studies are limited, some studies suggest that higher dietary tryptophan intake may help reduce symptoms like anxiety, irritability, and low mood – likely through enhanced levels of serotonin in the brain (85, 86). In mice, tryptophan-rich diets have been shown to improve depression and anxiety behaviors (86, 87).

Digestive enzymes can also serve as a targeted approach for some food sensitivities. Oral lactase, for example, decreases hydrogen levels and symptoms after lactose intake. Similarly, α-galactosidase, which breaks down galactooligosaccharides in legumes, nuts, and soy, has been shown to reduce symptoms in patients sensitive to plant-based protein sources (83).

Though food avoidance and use of exclusion diets can reduce symptoms for some patients, there is growing concern and some preliminary evidence that the use of exclusion diets can increase the risk for development of avoidant/restrictive food intake disorder (ARFID) among patients who follow these diets without sufficient provider guidance (88). Therefore, to reduce the risk of ARFID when using elimination diets, it is recommended that providers (e.g., gastroenterologists, dietitians) routinely monitor the extent of a patient’s food restriction, especially in those with anxiety or rigid eating behaviors. Screening for disordered eating patterns is essential, and involving a behavioral health specialist can provide the added support some patients need. Treatments like exposure-based CBT can gradually help patients feel more comfortable with eating, support better nutrition, and improve their overall quality of life (89). For screening, the Nine-Item ARFID Screen (NIAS) is a brief, validated tool that assesses three core domains of ARFID: sensory sensitivities, fear of aversive consequences, and low interest in eating (90). Using tools like the NIAS in conjunction with clinical judgment can help identify at-risk patients early and guide timely referral to specialized care.

In sum, dietary interventions are often best implemented under the supervision of a dietician, when possible, to mitigate against inadvertent impairment of nutritional status and/or eating-related quality of life.

## Emerging treatments

Outside of existing medications and therapies, there are emerging pharmacological and procedural treatments that have demonstrated promising results for the management of DGBIs, such as low dose naltrexone, transcutaneous vagal nerve stimulation (tVNS), and fecal microbiota transplant (FMT). Naltrexone is a peripherally restricted κ-opioid antagonist that has been used for chronic pain syndromes, and one open label study concluded that low dose naltrexone increased number of pain free days in IBS (91). tVNS via the auricular concha in animal models of IBS reversed both gastrointestinal permeability and depression-like symptoms (92), and human studies of tVNS have shown decreased intestinal permeability (93)and trends towards clinical remission in Crohn’s disease (94). FMT is another avenue of active investigation and promise given findings of intestinal dysbiosis in DGBI (95). A placebo controlled RCT of FMT in IBS showed improved IBS symptoms at 12 weeks, though the results diminished over a year (95). Available data in animal and human studies suggest these treatments have therapeutic potential for DGBI, but further robust studies are required before their widespread use.

## Approach to integrating pharmacotherapy, psychotherapy, and dietary interventions into treatment

**Figure 1:**
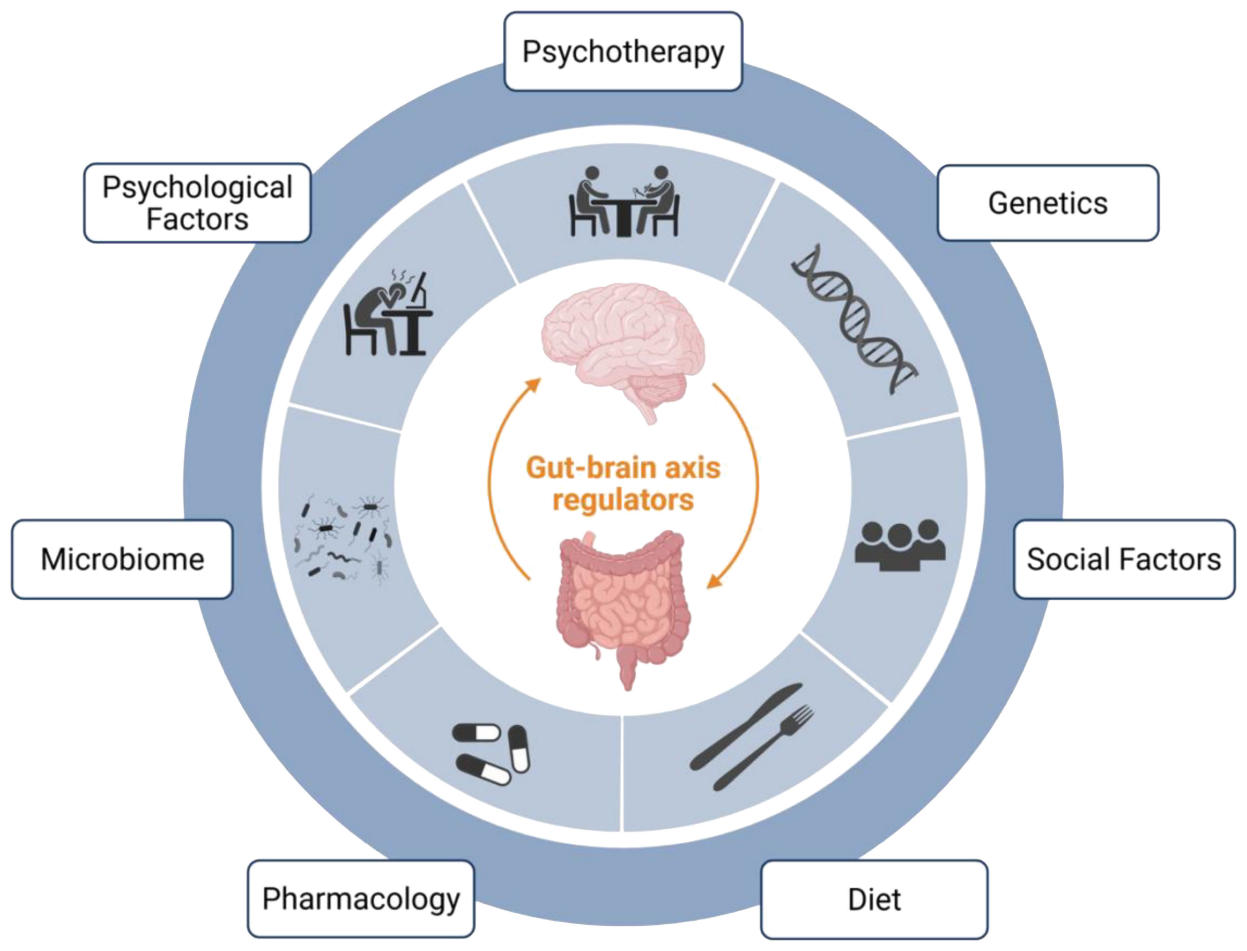
Select gut-brain axis regulators. This figure depicts some of the key regulators of DGBI symptomatology, each of which affects the gut-brain axis. Some of these factors are more easily modifiable (e.g., social factors, diet, microbiome, and psychological factors), and represent potential treatment targets. Finally, direct treatment strategies – which also modulate the gut-brain axis - include pharmacology and psychotherapy. Created in BioRender. Madva, EN. (52) https://BioRender.com/rl3j79f.

It can be helpful to have a systematic approach for when to integrate these treatment modalities (e.g., pharmacotherapy, psychotherapy, and dietary interventions) into the treatment pathway for each patient. While many systems and individual patient factors will likely impact treatment implementation in practice, we have found the approach suggested by Keefer et al. (65) to be quite helpful (65). This approach suggests using the patient’s most prominent presenting symptom (e.g., gastrointestinal symptom, psychiatric symptom, or behavior/anxiety specific to the gastrointestinal symptom) as a guide. Patients presenting primarily with a gastrointestinal symptom (e.g., nausea, pain) may benefit from earlier treatment with a neuromodulator, to at least quell some symptoms, prior to being referred for a BGBT, and to facilitate more successful BGBT engagement. Patients presenting primarily with a psychiatric symptom (e.g., depression, anxiety) often benefit from earlier referral to psychiatry, ideally for collaboration around selection of a neuromodulating agent. And finally, patients presenting primarily with anxiety or behaviors specific to their gastrointestinal illness, such as gastrointestinal symptom-specific anxiety or illness-specific coping behaviors, would likely benefit from earlier referral for a BGBT. The foundation of treatment for all patients, regardless of their presenting symptom, should likely include a combination of symptom-specific medical treatment, lifestyle modification, stress management, and dietary intervention (65) (**Figure 1**).

## Limitations of this study

This narrative review has several limitations. First, even though we developed several search strategies, the final selection of studies was conducted using an approach informed by the authors’ expertise, rather than a systematic methodology, which may have introduced selection bias. Second, publication bias may have influenced the body of literature considered, as studies with negative or null findings are less likely to be published. Third, the heterogeneity of outcome measures across studies—ranging from symptom severity to quality of life—limits the ability to directly compare findings or draw unified conclusions. Fourth, this review mostly focused primarily on IBS and FD due to data availability. As a result, these summarized findings may not be generalizable to other, less-studied DGBIs, such as functional biliary pain or centrally mediated abdominal pain syndrome. Finally, most of the literature reviewed is based on populations in Western, high-income countries, which may reduce the generalizability of conclusions to non-Western or resource-limited settings.

## Conclusion

In conclusion, there is a well described and robust connection between the gut and the brain. Dysfunction of the gut-brain connection can manifest as an array of psychiatric and gastrointestinal symptoms. As the DGBI are conceptualized as stress-sensitive biopsychosocial disorders, the most effective treatment approaches are comprehensive, aiming to address the biological, psychological, and social factors contributing to both the development and maintenance of these often-debilitating symptoms. There is a growing body of evidence supporting the use of gut-brain axis medications, BGBTs, and dietary therapies to target DGBI-related symptoms. In sum, a multidisciplinary treatment approach, that benefits from the expertise of gastrointestinal, mental health, and dietary clinicians, has the potential to significantly improve clinical outcomes.
