# Supplementary material for: A multidisciplinary approach to the management of disorders of gut-brain interaction: psychopharmacology, psychotherapy, and diet
Source: Front Gastroenterol (Lausanne). 2025 Oct 7;4:1637172. doi: 10.3389/fgstr.2025.1637172 (PMC12885561; doi:10.3389/fgstr.2025.1637172)
Supplement: Supplementary file 1 [file Table1.docx]

**SEARCH STRINGS**

**Neurophysiological Basis of Gastrointestinal Disorders (Brain-Gut Interaction)**

(("Disorders of gut-brain interaction"[tiab] OR "DGBI"[tiab] OR "functional gastrointestinal disorder*"[tiab] OR "functional GI disorder*"[tiab] OR "irritable bowel syndrome"[tiab] OR "IBS"[tiab] OR "functional dyspepsia"[tiab] OR "functional constipation"[tiab] OR "functional diarrhea"[tiab]))

AND

("brain-gut axis"[tiab] OR "gut-brain axis"[tiab] OR "visceral hypersensitivity"[tiab] OR "central sensitization"[tiab] OR "autonomic nervous system"[tiab] OR "hypothalamic-pituitary-adrenal axis"[tiab])

AND

("mechanism"[tiab] OR "neurobiology"[tiab] OR "pathophysiology"[tiab])

AND

("1995/01/01"[PDAT] : "3000"[PDAT]) AND English[lang]

AND

humans[MeSH Terms] AND ("adult"[MeSH Terms])

**Results: 111**

**Psychological Comorbidity in Gastrointestinal Diseases**

(("Disorders of gut-brain interaction"[tiab] OR "DGBI"[tiab] OR "functional gastrointestinal disorder*"[tiab] OR "functional GI disorder*"[tiab] OR "irritable bowel syndrome"[tiab] OR "IBS"[tiab] OR "functional dyspepsia"[tiab] OR "functional constipation"[tiab] OR "functional diarrhea"[tiab]))

AND

("anxiety"[ti] OR "depression"[ti] OR "mood disorder"[ti] OR "psychiatric comorbidity"[ti])

AND

("1995/01/01"[PDAT] : "3000"[PDAT]) AND English[lang] AND humans[MeSH Terms]

AND

("adult"[MeSH Terms])

**Results: 185**

**Pharmacology**

(("Disorders of gut-brain interaction"[tiab] OR "DGBI"[tiab] OR "functional gastrointestinal disorder*"[tiab] OR "functional GI disorder*"[tiab] OR "irritable bowel syndrome"[tiab] OR "IBS"[tiab] OR "functional dyspepsia"[tiab] OR "functional constipation"[tiab] OR "functional diarrhea"[tiab]))

AND

("pharmacotherapy"[tiab] OR "neuromodulator*"[tiab] OR "tricyclic antidepressants"[tiab] OR "SSRIs"[tiab] OR "SNRI"[tiab] OR "antispasmodics"[tiab] OR "gut-brain neuromodulators"[tiab])

AND

("1995/01/01"[PDAT] : "3000"[PDAT])

AND

English[lang]

AND

humans[MeSH Terms]

AND

("adult"[MeSH Terms])

**Results: 113**

**Psychotherapy**

(("Disorders of gut-brain interaction"[tiab] OR "DGBI"[tiab] OR "functional gastrointestinal disorder*"[tiab] OR "functional GI disorder*"[tiab] OR "irritable bowel syndrome"[tiab] OR "IBS"[tiab] OR "functional dyspepsia"[tiab] OR "functional constipation"[tiab] OR "functional diarrhea"[tiab]))

AND

("cognitive behavioral therapy"[tiab] OR "CBT"[tiab] OR "gut-directed hypnotherapy"[tiab] OR "mindfulness"[tiab] OR "psychological therapy"[tiab] OR "psychotherapy"[tiab] OR "brain-gut behavior therapy"[tiab] OR "behavioral intervention"[tiab])

AND

("1995/01/01"[PDAT] : "3000"[PDAT])

AND

English[lang]

AND

humans[MeSH Terms]

AND

("adult"[MeSH Terms])

**Results: 180**

**Dietary Interventions**

**(("Disorders of gut-brain interaction"[tiab] OR "DGBI"[tiab] OR "functional gastrointestinal disorder*"[tiab] OR "functional GI disorder*"[tiab] OR "irritable bowel syndrome"[tiab] OR "IBS"[tiab] OR "functional dyspepsia"[tiab] OR "functional constipation"[tiab] OR "functional diarrhea"[tiab]))**

**AND**

**("FODMAP"[tiab] OR "elimination diet"[tiab] OR "dietary therapy"[tiab] OR "nutrition intervention"[tiab] OR "probiotic supplementation"[tiab] OR "prebiotic supplementation"[tiab])**

**AND**

**("1995/01/01"[PDAT] : "3000"[PDAT])**

**AND**

**English[lang]**

**AND**

**humans[MeSH Terms]**

**AND**

**("adult"[MeSH Terms])**

**Results: 217**
